# Regulation of plant cell wall degradation by light in *Trichoderma*

**DOI:** 10.1186/s40694-018-0052-7

**Published:** 2018-04-24

**Authors:** Monika Schmoll

**Affiliations:** Center for Health and Bioresources, AIT Austrian Institute of Technology GmbH, Konrad Lorenz Straße 24, 3430 Tulln, Austria

**Keywords:** *Trichoderma reesei*, *Hypocrea jecorina*, CAZymes, Light response, Signal transduction, Surface sensing, Carbon source utilization, EMSA, Genomic clusters

## Abstract

*Trichoderma reesei* (syn. *Hypocrea jecorina*) is the model organism for industrial production of plant cell wall degradating enzymes. The integration of light and nutrient signals for adaptation of enzyme production in *T. reesei* emerged as an important regulatory mechanism to be tackled for strain improvement. Gene regulation specific for cellulase inducing conditions is different in light and darkness with substantial regulation by photoreceptors. Genes regulated by light are clustered in the genome, with several of the clusters overlapping with CAZyme clusters. Major cellulase transcription factor genes and at least 75% of glycoside hydrolase encoding genes show the potential of light dependent regulation. Accordingly, light dependent protein complex formation occurs within the promoters of cellulases and their regulators. Additionally growth on diverse carbon sources is different between light and darkness and dependent on the presence of photoreceptors in several cases. Thereby, also light intensity plays a regulatory role, with cellulase levels dropping at higher light intensities dependent in the strain background. The heterotrimeric G-protein pathway is the most important nutrient signaling pathway in the connection with light response and triggers posttranscriptional regulation of cellulase expression. All G-protein alpha subunits impact cellulase regulation in a light dependent manner. The downstream cAMP pathway is involved in light dependent regulation as well. Connections between the regulatory pathways are mainly established via the photoreceptor ENV1. The effect of photoreceptors on plant cell wall degradation also occurs in the model filamentous fungus *Neurospora crassa*. In the currently proposed model, *T. reesei* senses the presence of plant biomass in its environment by detection of building blocks of cellulose and hemicellulose. Interpretation of the respective signals is subsequently adjusted to the requirements in light and darkness (or on the surface versus within the substrate) by an interconnection of nutrient signaling with light response. This review provides an overview on the importance of light, photoreceptors and related signaling pathways for formation of plant cell wall degrading enzymes in *T. reesei*. Additionally, the relevance of light dependent gene regulation for industrial fermentations with *Trichoderma* as well as strategies for exploitation of the observed effects are discussed.

## Background

The continuing alteration between light and darkness on earth caused by its rotation resulted in an evolutionary adaptation of the majority of living beings to day and night. The physiological changes connected to day and night are triggered by the circadian clock, which governs preparation to the upcoming day or night. Exposure to light at unexpected times causes phase shifts in the clock controlled cycles and hence alters the connected gene regulation. Nevertheless, control by the circadian clock and response to changing light conditions are distinct processes [1]. This adaptation does not only concern obvious necessities such as the protection against harmful UV light during the day or dealing with higher temperatures and lower humidity levels connected to sunlight versus darkness. Also, the adjustment of metabolic processes to light and darkness has evolved—not only in humans, but also in fungi [2, 3],—which raises the question whether fungi do have an equivalent to the physiological condition of sleep or the physiologically different situation in day and night in humans.

In fungi, light is highly relevant to diverse physiological regulation mechanisms and impacts many signaling pathways that integrate light response with metabolism, stress response and development [4]. Even though many of the fungi currently investigated in academia and industry never experience natural conditions of day and night, their gene expression levels and physiology still follow circadian rhythms [5], which also target metabolism [6–8]. Groundbreaking work in elucidation of the molecular machinery governing circadian rhythmicity and light response has been done in *Neurospora crassa* [9–11], which involves transcriptional cascades as well as epigenetic and posttranslational modifications [12, 13] and many of the studies on light effects in dthe fungi built on the discoveries from *N. crassa* thereafter.

*Trichoderma reesei* represents one of the most important filamentous fungi nowadays used in industry for production of homologous and heterologous enzymes—predominantly for biofuel production [14, 15]. *T. reesei* expresses diverse carbohydrate active enzymes (CAZymes), the most important being cellulases and hemicellulases [16]. Induction of these enzymes occurs on different carbon sources such as cellulose or lactose or in the presence of sophorose, but also on other carbon sources representing building blocks of plant cell wall material [17, 18]. Repression occurs on easily metabolizable carbon sources like glucose by the function of carbon catabolite repression. Secondary metabolism has not been studied in detail in *T. reesei* yet, but harmful mycotoxins are not known to be produced by this fungus [19]. *T. reesei* was the first industrially relevant filamentous fungus for which the method of sexual crossing became available. Sexual development is dependent on specific conditions of light, temperature and carbon source in the medium and numerous regulators, including the *T. reesei* photoreceptors and several signaling compounds [20, 21].

**Fig. 1:**
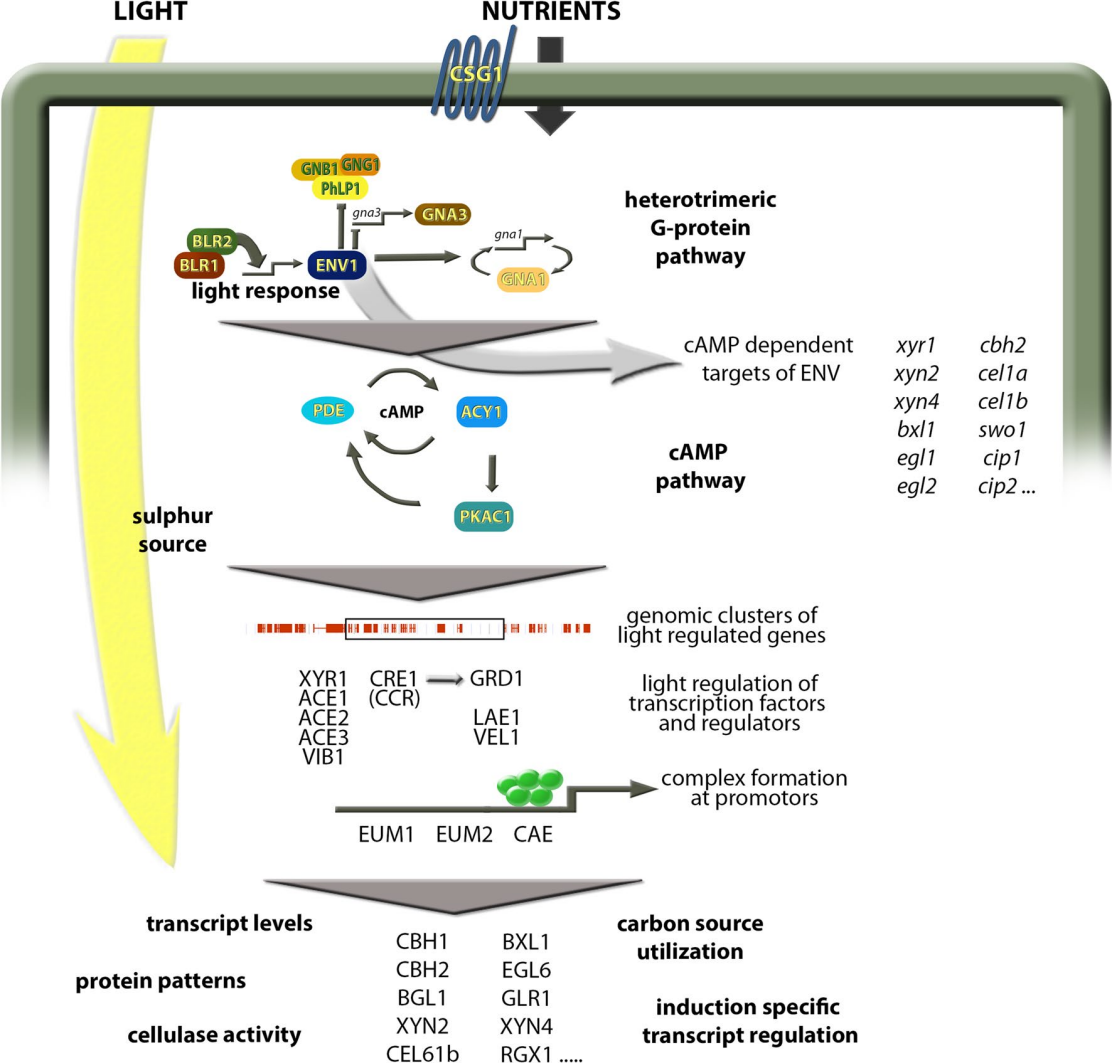
Schematic overview of pathways involved in light regulation of plant cell wall degradation

**Fig. 2:**
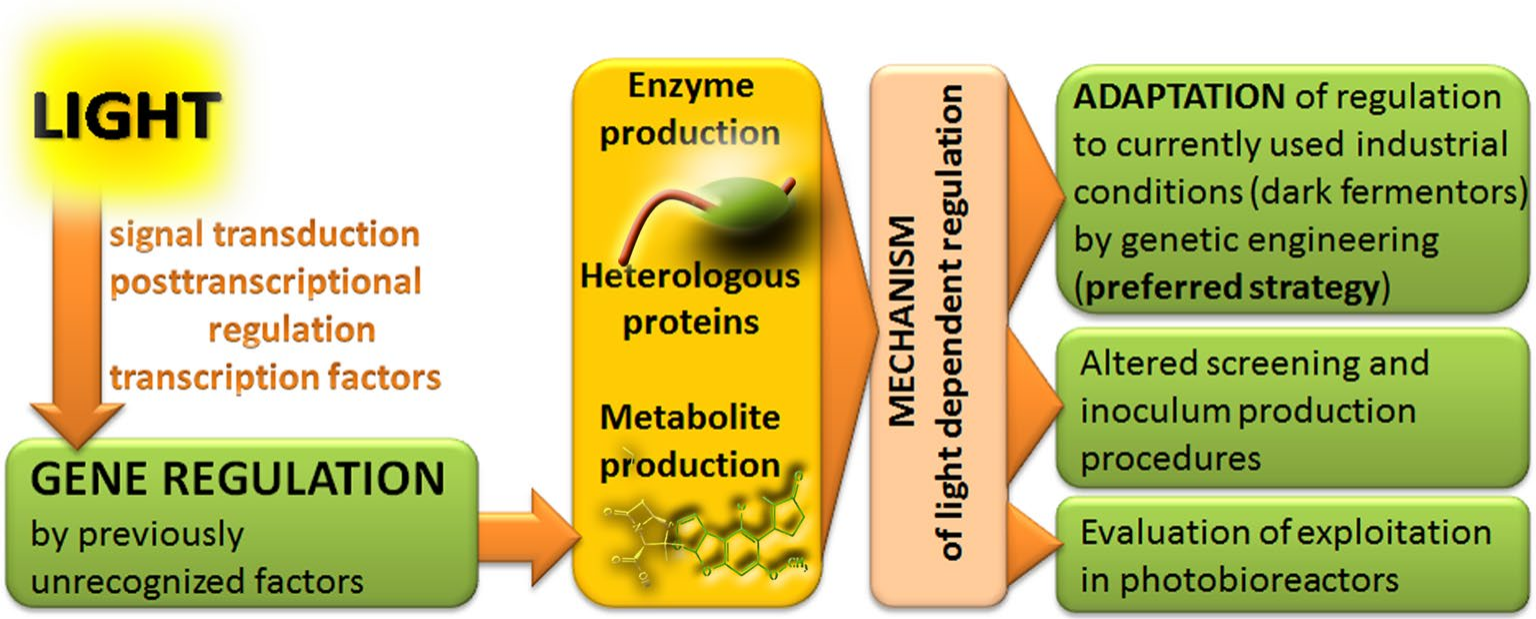
Schematic representation of strategies for strain improvement by exploiting of light dependent effects. Gene regulation in *T. reesei* is considerably influenced by light, with the light signal coordinated with nutrient signals via the signal transduction pathways of heterotrimeric G-proteins and cAMP signaling. Posttranscriptional regulation of cellulase gene expression is triggered by a G-protein coupled receptor, whose signal is channeled through the G-protein pathway and its subunits. The consequences of light exposure include changes in normal enzyme production, production of heterologous proteins and secondary metabolites expressed from homologous or heterologous gene clusters. Once the mechanisms of light dependent regulation are understood, this information can serve to improve performance under currently applied industrial conditions (dark fermentors) by knowledge based genetic engineering. Additionally, screening procedures are recommended to be performed under controlled light conditions and inoculum production can be improved. For high value products, illumination or specifically applied light regimes in photobioreactors can be evaluated

In this review, I will first give a short overview on composition of plant cell walls and their degradation followed by a general introduction to the light response pathway and known physiological effects of light on fungi in order to familiarize the reader with the two topics connected in this review. Thereafter I briefly describe the discovery of the influence of light on cellulase regulation along with some general findings on the topic later on. Subsequently, I explain the impact of light and the light response machinery on regulatory pathways influencing metabolic functions in *Trichoderma* with an emphasis on plant cell wall degradation and interconnections between nutrient and light signaling pathways (Fig. 1). Individual regulatory factors as well as their genome/transcriptome wide effects in dependence of light will be discussed including alterations on different carbon sources, non random distribution of light regulated genes and the interplay of light response with carbon catabolite repression. Based on that, light dependent differences at the promoter level and in carbon source utilization are outlined. Finally, the relevance of light for regulation of specific, known factors crucial for highly efficient plant cell wall degradation as well as aspects of light influenced processes for research and industry (Fig. 2) are discussed.

## The plant cell wall and the enzymes degrading it

Lignocellulosic plant biomass represents the most important carbon source on our planet and it is hence of high ecological importance as major component in the global carbon cycle. Plant biomass is composed of several polymers including the recalcitrant cellulose, hemicellulose and lignin, but also pectin and starch [22]. Filamentous fungi are highly efficient degraders of plant biomass and have evolved a complex yet very efficient machinery for degradation of plant cell walls [23, 24]. The involved Carbohydrate Active enZymes (CAZys) act on glycoside linkages in the plant cell wall polysaccharides and can have a broad range of activities [25, 26]. It is assumed that glycoside hydrolases, the most important enzymes for plant cell wall degradation, have evolved from a common ancestor. In the course of the subsequent specialization, saprotropic fungi like *T. reesei* appear to have lost a significant number of genes including glycoside hydrolases [27]. Since cellulases are usually found coregulated, the cellobiohydrolases CBH1/Cel7a and CBH2/CEL6a are frequently used as representatives for cellulase expression. In recent years, the oxidation of cellulose by polysaccharide monooxygenases (previously assigned to glycoside hydrolase family 61) was shown to significantly contribute to plant cell wall degradation [28, 29]. Interestingly, an involvement of light and photosynthetic pigments in oxidation of polysaccharides was postulated [30].

Regulation of plant cell wall degrading enzymes is complex and occurs at the transcriptional level involving a plethora of transcription factors and regulators, some of the most important being XYR1, ACE1, ACE2, ACE3, VIB1 and the HAP complex [23, 31, 32]. Recently, also a post-transcriptional section of cellulase regulation was shown [33]. Thereby, a considerable number of substrates cause induction of expression of plant cell wall degrading enzymes [16–18]. Importantly, the mechanism of carbon catabolite repression, with its major transcription factor CRE1 serves to avoid biosynthesis of enzymes in the presence of easily metabolizable carbon sources [23, 34]. By application of their sophisticated regulatory machineries, fungi adjust the amount and type of enzymes to produce to their environment—be it a tropical forest or an industrial fermentor.

## The light response pathway in *Trichoderma* spp.

*Trichoderma* spp. have a long tradition of research towards fungal light responses and their physiological consequences [35, 36]. Eight proteins are considered to be responsible for light perception [36] including BLR1 and BLR2 (blue light regulators 1 and 2): two GATA-type transcription factors predominantly acting as a photoreceptor complex, the photoreceptor ENV1, two photolyases, a cryptochrome, a phytochrome and an opsin, which is however only present in the genome of *Trichoderma atroviride*, but not in *T. reesei* [36, 37].

The major blue light photoreceptors BLR1 and BLR2, which are the homologues of *N. crassa* WC-1 and WC-2 (white collar 1 and 2) [38], were first characterized in *T. atroviride*, where they are essential for blue light induced conidiation [39]. Thereafter, ENV1, a homologue of *N. crassa* VVD [40] and the third photoreceptor was detected in *T. reesei* and an unexpectedly broad range of physiological functions in light response, development, stress response and metabolism was shown for ENV1 that exceed those of BLR1 or BLR2 [41, 42]. In contrast to *T. atroviride*, deletion of *blr1* or *blr2* does not abolish conidiation in *T. reesei* [43, 44], while lack of ENV1 causes a severe growth phenotype in light [42], which does not occur on every carbon source [45, 46]. In both fungi, BLR1 and BLR2 exert common as well as independent functions and also influence gene expression in darkness [46, 47]. Consequently, while there is considerable overlap in functions, there are also some differences in the light response machinery in *Trichoderma* spp. which may cause altered responses.

BLR1 and BLR2 are GATA type zinc finger transcription factors and are assumed to act in a complex. BLR1 contains three PAS domains, and a PAS/LOV domain, which is responsible for reception of the light signal due to the flavin moiety bound in this domain. BLR2 contains only one PAS domain and ENV1 contains a PAS/LOV domain like BLR1 [36]. Light dependent induction of *env1* is strictly dependent on the presence of both BLR1 and BLR2 [43, 48, 49]. Nevertheless, phenotypic effects of deletion of *blr1* or *blr2* are much less severe than deletion of *env1* in light [41–44, 46].

The hierarchy in the light response cascade thereby depends on the function of the respective regulators in *T. reesei*. In many cases, ENV1 acts via BLR1 and BLR2, for example with respect to growth and sexual fertility [48]. Like BLR1 and BLR2, also ENV1 has individual functions [46], albeit often effects reminiscent of photoadapation occur: If the negative effect of ENV1 is relieved by its deletion, the usually balancing positive effect of BLR1 and BLR2 becomes obvious [48]. This balanced function of ENV1, BLR1 and BLR2 further explains the rescue of the severe growth phenotype of strains lacking ENV1 in light in ∆*env1* double or triple mutants with *blr1* and/or *blr2* deletions [48].

In *T. atroviride*, enhanced expression of *blr2* causes increased photoconidiation and higher transcript levels of light induced genes, while the opposite effect is caused by overexpression of *blr1*. *Blr2* overexpression further causes higher sensitivity to blue light and complex formation of BLR1/BLR2 is assumed to be required for appropriate light perception in *T. atroviride* [50].

## Physiological responses to light in *Trichoderma*

Life in soil—a mainly dark environment—and on the surface of a substrate are fundamentally different in terms of UV radiation, oxygen, temperature stability, humidity and levels of reactive oxygen species (ROS). Also during day and night many of these environmental properties change and hence, fungi evolved to use light as a fast indicator of the expected changes and prepare for daily alterations by applying circadian rhythms [51].

Blue light responses in *T. reesei* include enhanced conidiation [43] and sexual reproduction [21, 52], secondary metabolism [46, 53, 54], growth [45] and altered regulation of enzyme gene transcription [46, 53], expression and activity [55]. Genes of the light response machinery including *phr1* encoding a photolyase, the photoreceptor gene *env1* and *frq*, which is required for circadian rhythmicity in *N. crassa*, the MAPkinase gene *tmk3* [45] as well as several genes involved in sexual development are light dependently regulated irrespective of the carbon source [33].

The earliest response to light evaluated in *T. reesei* was after 15 min [45], albeit much shorter light pulses are assumed to be sensed and can cause altered gene regulation. Thereby it has to be considered that not only the duration but also the light intensity (fluence rate) is important [55]. An effect of red light on the regulation of few genes was detected for *T. atroviride* [56], while in *T. reesei* no effect of red light was observed so far [43].

In *T. atroviride*, light stimulates the tolerance to osmotic stress through the Hog1-related MAPkinase TMK3. This stimulation of stress signaling pathways by light is considered a benefit for the cell [57]. Interestingly, *T. reesei* TMK3 was found to regulate cellulase production [58, 59], albeit an influence of light was not tested in this study. Together these findings indicate that the MAPkinase pathway contribute to light dependent modulation of cellulase gene expression.

In *T. reesei* an involvement of ENV1 in stress response was observed, which requires the conserved amino acids C96 and T101 [60, 61]. Thereby, C96 is evolutionarily conserved in Hypocreaceae, which in contrast to for example *N. crassa*, indicating the integration of stress and light responses via ENV1.

## An unexpected effect of light and photoreceptors on cellulase gene expression

Traditionally, filamentous fungi were grown under random light conditions, except for investigation of circadian rhythms or photoreceptor functions in *N. crassa*. *Trichoderma* spp. also served as models for analysis of light responses in the 1970s and 80s [36], but this did not lead to cultivation under controlled light conditions thereafter because no connection to carbon metabolism was assumed.

A screening assay for genes differentially expressed between the cellulase negative mutant strain QM9978 and the early high production mutant QM9414 surprisingly yielded the photoreceptor gene *env1* as differentially regulated between the two strains under inducing conditions (cellulose and sophorose) [62]. ENV1 is a homologue of the *N*. *crassa* photoreceptor VIVID (VVD), which negatively acts on the photoreceptor complex established by WC-1 and WC-2, the homologues of BLR1 and BLR2 [63, 64]. However, *T. reesei env1* could not complement a *N. crassa vvd* non-functional mutant and is hence not considered a functional homologue [42]. Still, subsequent research revealed a considerable functional overlap of ENV1 and VVD.

Consequently, cultivation of *T. reesei* was performed under controlled light conditions and with the cellulase inducing carbon sources cellulose and lactose in order to assess a potential effect of light on enzyme expression [42]. Indeed, striking differences in cellulase transcript levels between growth in light and in darkness were observed upon growth on cellulose, which did not correlate with previous results in a conventional incubator with random light pulses—neither in light nor in darkness. Transcript levels of *cbh1* increased by roughly 50% upon growth in constant light on cellulose and an important function of ENV1 in this regulation became obvious, with decreased *cbh1* levels on lactose, transient increase on cellulose in darkness and decrease in light in the mutant strain [42]. Upon induction of cellulase gene expression by the natural inducer sophorose, we found that in darkness, cellulase transcript levels increase with time, while in light cellulase induction appears to be accelerated, but transient [65].

## Functions of the light response machinery in cellulase regulation

Searching for the basis of light dependent regulation of cellulase genes, comparison of the promoters of *env1* and *vvd* revealed two potential DNA binding motifs, EUM1 (envoy upstream motif; 5′ CTGTGC 3′) and EUM2 (5′ ACCTTGAC 3′). EUM1 is also present in the promoters of the cellulase genes *cbh1* and *cbh2* as well as in the promoters of *blr1* and *blr2* suggesting a potential for co-regulation or feedback [42] (see also below). Additionally, the same study revealed that *env1* is not expressed in the cellulase negative strain QM9978, which has a mutation in the EUM1 promoter motif in the *env1* promoter. However, complementation with the entire *env1* gene that included this promoter region did not rescue cellulase expression in QM9978 [42]. EUM1, light responsive elements (LREs) [66] and GATA sites, which all might be predictive for binding of the photoreceptor complex or other light regulatory factors, were found in numerous promoters of known cellulose and hemicellulose degrading enzymes [67].

Later on, investigation of the genome of QM9978 and comparison to that of QM6a recently led to the finding that the PhoG homologue VIB1 is responsible for the defect in cellulase induction of this strain. A translocation of the gene abolished expression of *vib1* in QM9978 [68]. Interestingly, *vib1* is strongly down-regulated in light upon growth on cellulose in the wild-type strain QM6a (Stappler and Schmoll, unpublished). Downregulation of *vib1* in light is further observed upon growth on lactose or sophorose [33]. Transcriptome analysis upon growth on cellulose showed that *vib1* is strongly regulated by ENV1 in light and by the adenylate cyclase ACY1 in light [33, 49]. Consequently, *vib1* is a cAMP dependent target of the light response pathway [49], which is also in agreement with the finding of the gene encoding ENV1 as a regulator of *vib1* to be down-regulated in QM9978 compared to QM9414 under inducing conditions [42] [62].

BLR1 and BLR2 positively regulate transcript levels of *cbh1* in *T. reesei* [43] and in *T. atroviride* [69]. Interestingly, effects are obvious in light and darkness, indicating that these photoreceptors also have a function in darkness in *T. reesei* [43, 46]. Accordingly, effects of ENV1, BLR1 and BLR2 were also observed in largely dark fermentor cultivations in *T. reesei*. Under these conditions, ∆*env1* secretes a more efficient enzyme mixture and for BLR2 an effect on secretion capacity was found, which leads to increased cellulase activities as well. ∆*blr1* forms more biomass, but its lower secretion capacity counteracts its efficiency in production of cellulose degrading enzymes [67].

Abundance of secreted proteins is considerably altered in light [55] including such important enzymes as CBH1, BGL1, XYN2 or CEL61B. BLR1 and BLR2 have a major impact in this light dependent regulation. While initial analyses on light response of cellulase regulation were performed in the QM9414 strain background, we later found significant differences between light and darkness also for QM6a [55], RutC30 and industrial production strains as well (unpublished results). Thereby, QM6a shows a much lower light tolerance than QM9414 in terms of alteration of cellulase gene expression. In QM9414 *cbh1* transcript levels initially increase with increasing light intensities and only drop at 5000 lx, while in QM6a already low light intensities abolish *cbh1* transcription. In ∆*blr1* and ∆*blr2*, this drop at high light intensities does not occur [55].

In addition to the photoreceptors, further regulators involved in light dependent signaling were identified as cellulase regulators. In *Aspergillus nidulans*, VeA and LaeA coordinate the light signal with fungal development and secondary metabolism [70]. Their homologues in *T. reesei*, VEL1 and LAE1 are important regulators of cellulase gene expression [71, 72]. Unfortunately, the latter studies were done under uncontrolled light conditions and hence a light dependent relevance of this regulation is not known. Since the function of VEL1 in development shows light dependent differences and a connection to photoreceptors [73, 74], this can also be expected for cellulase regulation.

## Signaling pathways involved in light dependent modulation of cellulase gene expression

For the presence of cellulose to be sensed in the environment, low molecular weight degradation products liberated from degradable plant material are likely signals that may be sensed by membrane bound receptors. Alternatively, an inducer synthesized outside the cell may be taken up via diffusion or a permease and initiate cellulase formation by an intracellular process. Both hypotheses are likely to describe contributions to the regulation of cellulase gene expression.

### The heterotrimeric G-protein pathway

The prime candidate pathway for sensing and transmission of an extracellular cellulose related signals is the heterotrimeric G-protein pathway. Signal transduction via the heterotrimeric G-protein pathway starts with reception of a signaling compound by a G-protein coupled receptor (GPCR). Once such a ligand binds to the GPCR, the heterotrimeric G-protein complex composed of G-protein alpha, beta and gamma subunits, dissociates, GDP is exchanged for GTP on the G-alpha subunit for activation and all subunits then modulate their target pathways [37, 75, 76].

*T. reesei* has 50 GPCRs, 3 G-alpha subunits (GNA1, GNA2 and GNA3), one G-beta subunit (GNB1) and one G-gamma subunit (GNG1). Additionally, two phosducin like proteins (PhLP1 and PhlP2), which are assumed to act as co-chaperones for G-protein beta and gamma folding are encoded in the genome as well as 7 RGS (regulator of G-protein signaling) domain proteins [37]. The G-protein alpha subunits GNA1 and GNA3 impact cellulase gene expression and this function is dependent on light [77, 78]. Constitutive activation of GNA3 led to a strong increase in cellulase transcripts upon growth on cellulose (around tenfold), but only in light. In darkness, neither constitutive activation nor knock down altered cellulase transcript levels [77]. Interestingly, upregulation of *gna3* without constitutive activation caused somewhat increased cellulase levels in light, which however did by far not reach the strong up regulation seen for the constitutive activation of GNA3. It is therefore likely that the rate of inactivation of the alpha subunit by the intrinsic GTPase of GNA3 plays a role. This function is enhanced by RGS (regulator of G-protein signaling) proteins, which are consequently assumed to be involved in cellulase regulation in *T. reesei* [77]. Of those, *rgs1* represents a light independent regulatory target of GNB1, GNG1 and PhLP1 [53].

Lack of GNA1 causes a strong increase of cellulase gene expression in darkness and abolishment in light upon growth on cellulose. Constitutive activation of GNA1 caused—like for GNA3—an increase in cellulase transcript levels in light [78]. For both GNA1 and GNA3, constitutive activation did not enable inducer independent cellulase gene expression. Consequently, the signal for induction of cellulase gene expression is separate from that transmitted by GNA1 and GNA3 and may indeed involve intracellular detection of an inducer.

The G-protein beta and gamma subunits GNB1 and GNG1 as well as the phosducin-like protein PhLP1, which is assumed to act as a chaperone for complex formation of GNB1 and GNG1, influence regulation of several glycoside hydrolases including *cbh1* and *cbh2* [53]. Cellulase activity increases in all three mutants, while transcript abundance of several genes encoding plant cell wall degrading enzymes decreases, indicating posttranscriptional regulation [53]. However, the light dependent effects of GNB1, GNG1 and PhLP1 are considerably less pronounced than the strongly positive impact of the G-alpha subunits GNA1 and GNA3 [53]. In summary, these findings are in line with a positive effect of phosducin-like proteins on the efficiency of G-protein signaling [79].

Only recently, a new aspect was added to the function of the heterotrimeric G-protein pathway in cellulase regulation. The class XIII of G-protein coupled receptors (GPCRs) was found to have only a minor influence on cellulase transcript levels (up to 50%), but both were required for normal specific cellulase activities on lactose. In the absence of CSG1, representing one of the two class XIII GPCRs of *T. reesei*, secreted activity levels even decreased to basal levels upon growth on cellulose and lactose [33]. Hence it is assumed that the signal received by CSG1 is responsible for posttranscriptional regulation of cellulase gene expression, a mechanism previously not known to be involved. Based on the light dependent function of GNA1 and GNA3 it can be assumed that these downstream subunits integrate the light dependent relevance with the signal received by CSG1 to a physiological response adapted to the change of day and night.

### The cAMP pathway

Cyclic adenosine monophosphate (cAMP) is one of the most important secondary messengers in living organisms. cAMP levels function as coincidence detectors due to their regulation by diverse environmental cues that are integrated to a defined level, which determines the extent of the effect on downstream pathways. Adenylate cyclase synthesizes cAMP and phosphodiestereases, which are activated by the presence of cAMP [80, 81], degrade it [82], forming a feedback cycle for adjustment of levels [80, 81]. Protein kinase A is cAMP dependent and consists of catalytic and regulatory subunits [37]. Light dependent effects on cAMP levels and adenylate cyclase activity have been shown previously for *Trichoderma* [83–85].

The cAMP dependent protein kinase A plays an important role in regulation of light responses in *T. atroviride* [86]. In *T. reesei*, the cAMP pathway is one of the targets of the heterotrimeric G-protein pathway and cAMP levels are impacted by both GNA1 and GNA3 [77, 78]. Investigation of the function of adenylate cyclase (ACY1) and the catalytic subunit 1 of protein kinase A (PKAc1) showed a light dependent effect on cellulase gene expression in both cases upon growth on lactose [87]. Deletion of *acy1* and *pkac1* caused increased light responsiveness of cellulase genes i.e. strongly increased differences between cellulase levels in light and darkness compared to the light dependent regulation in the wild-type. Regulation of the transcript levels of the transcription factors ACE1 and ACE2 that regulate transcription of cellulase genes show a response to light, but do not correlate well with cellulase levels. Modification of transcript levels of the cellulase regulator XYR1 strongly resembles the pattern of regulation seen for cellulase genes. Hence, it is assumed that the cAMP pathway does not directly target phosphorylation of XYR1, but rather causes phosphorylation of a regulator of XYR1, which by modification of XYR1 acts on cellulase transcription [87].

In this respect it is interesting that deletion of *acy1* or *pkac1* also causes decreased levels of the photoreceptor gene *env1* [87], which regulates cellulase levels [42]. Phosphorylation of the photoreceptor complex by protein kinase A was shown in *N. crassa* [88] and critically impacts the function of the circadian clock by establishing a negative feedback loop. A similar mechanism may be responsible for regulation of *env1* levels in *T. reesei*. In turn, strains lacking ENV1 show strongly decreased cAMP levels, which is attributed to an impact of ENV1 on the function of phosphodiesterases rather than adenylate cyclase [89]. Such a mechanism would be in agreement with a feedback mechanism for fine-tuning of the integration of light response with nutrient signaling. Only recently we could confirm the involvement of a phosphodiesterase in light dependent cellulase regulation and the connection to ENV1 (E. Stappler et al., manuscript in preparation).

### Interconnections between light response machinery and nutrient signaling pathways

The finding that light and its photosensors are involved in regulation of cellulase gene expression, indicated that nutrient utilization is interrelated with light response. The findings of the influence of the light signaling pathway on carbon-compound and carbohydrate metabolism [53] further raised the question as to the interconnections between nutrient signaling and sensing light. More detailed investigation of the heterotrimeric G-protein pathway and the cAMP pathway indicated that the light signal must be integrated with the transmitted nutrient signals.

BLR1 and BLR2 do not have a major impact on the G-protein signaling pathway [49]. ENV1 negatively influences *blr1* and *blr2* transcript levels, which in turn are required for induction of *env1* in light. This mutual regulation causes a steady state level of transcripts for these three genes [49]. ENV1 is required for the positive feedback cycle of GNA1, which increases *gna1* transcript levels upon constitutive activation. In turn, GNA1 negatively regulates *env1* transcript levels [89]. Concerning the regulation mechanism of GNA3/*gna3*, ENV1 is not involved in establishing the *gna3* feedback cycle, but negatively regulates *gna3* transcript levels in light [89]. During investigation of the mutual regulatory effects of BLR1, BLR2 and ENV1 as well as GNA1, GNA3, GNB1, GNG1 and PhLP1, a pair with regulatory interaction at early light response emerged: PhLP1 and ENV1 [49]. With its negative effect on transcript levels of *phlp1*, *gnb1* and *gng1*, ENV1 dampens G-protein signaling during early light response, presumably to provide resources for protection from light. Thereafter, PhLP1 can exert a positive effect on complex formation of GNB1 and GNG1 and G-protein signaling in general, which is likely to be important for metabolic adaptation to light [49]. The striking overlap in regulatory targets of photoreceptors, G-protein pathway components and ACY1 suggests that this interrelationship constitutes a core function in adaptation beyond transient effects in early light response.

Surprisingly, these interconnection studies revealed that it is not the photoreceptor complex comprising the two BLR proteins, but rather ENV1 that acts in a central position as a checkpoint, which is in line with its strong effect on gene regulation in light [46]. The more prominent role of ENV1 in comparison to BLR1 and BLR2 is also obvious in the role of ENV1 in the regulation of other physiological processes such as sexual development or stress response [41].

The strikingly low cAMP levels in mutants lacking ENV1 indicated that ENV1 could exert at least part of its function via the cAMP pathway adding another link between light response and nutrient signaling (see also above). The ∆*acy1* strain showed a striking overlap of regulated genes in light, but not in darkness [49]. These genes have in common their regulation in the presence of strongly decreased or abolished cAMP levels. Among these cAMP targets there are numerous glycoside hydrolases, auxiliary proteins of cellulose degradation as well as the cellulase and hemicellulase regulator gene *xyr1* [49]. Functional category analysis of these genes showed a strong enrichment in C-compound and carbohydrate metabolism. Consequently, the regulatory function of ENV1 in targeting regulation of enzyme expression is to a considerable extent mediated by its effect on cAMP levels [49]. Interestingly, many of the genes down-regulated in ∆*env1* and ∆*acy1* in the light are also down-regulated on soluble carbon sources compared to the insoluble carbon source cellulose [33]. Consequently, the cAMP dependent output of the regulatory cascade triggered by ENV1 could be targeted at sensing and/or reaction to a surface of specifically to cellulose, but not to a soluble carbon source [33].

In summary, ENV1 emerged as a major checkpoint between nutrient and light signaling [41]. However, it still remains to be shown whether this interrelationship is established directly by protein–protein interaction and an influence on activity of the targeted regulators or if the influence is indirect by an impact on regulation of abundance and/or activity of signaling factors, for example kinases, which in turn target regulatory factors. The considerable impact of deletion of *env1* on the transcriptome in light [46] makes the latter hypothesis more likely.

## Light dependent regulation of cellulose degradation as influenced by the sulphur source

Genes involved in metabolic processes are major targets of light response in *T. reesei* [46, 53]. Amino acid metabolism and sulphur metabolism are among these targets [33, 46, 53] and these functional categories are further found correlated with altered levels of cellulase production and carbon catabolite repression [90–92].

Studies aimed at identification of regulators binding to CAE within the *cbh2* promoter revealed the sulphur related regulator protein LIM1, a homologue of *N. crassa* SCON2, as a candidate regulator [93]. Investigation of expression of *lim1* under cellulase inducing conditions (cellulose, sophorose) in light and darkness supported this hypothesis. Interestingly, in the absence of a sulphur source, the methionine content, which can be used as a sulphur source, was important for cellulase gene expression and high sulphur conditions (5 mM) were even deleterious for growth in light. In the presence of normal sulphur levels in the MA minimal medium on cellulose, the same amount of methionine causes an increase of cellulase gene expression in darkness, but abolishment of *cbh1* transcription in light. Therefore, methionine appears to represent a signal molecule with a light dependent impact on cellulase regulation [93].

Additionally, sulphate uptake upon growth on cellulose is regulated by light, while this is not the case with glucose as carbon source [93]. Hence, light dependence of sulfur metabolism is dependent on the carbon source.

Interestingly, we found a significant enrichment (*p* value > 0.05) of genes involved in amino acid metabolism among those specifically down-regulated under cellulase inducing conditions [33]. Thereby, significant enrichment in methionine metabolism was detected only in darkness [33]. Moreover, significant enrichment in functions in methionine metabolism and sulphur metabolism were detected for down-regulated genes in ∆*env1* on cellulose [46]. Only recently, metabolic modelling of *T. reesei* under chemostat conditions with controlled growth rate revealed sulphur assimilation as a major limiting factor of protein production [94].

## Light dependent gene regulation patterns in *Trichoderma* spp.—a genome wide view

First data on the influence of light on the transcriptome showed regulation of 2.8% of genes in response to light in *T. atroviride* [56]. In *T. reesei,* a comparable extent of light dependent gene regulation (2.7%) was detected in the early high cellulase expression mutant QM9414 upon growth on cellulose [53]. The gene set up-regulated upon growth in light on cellulose is significantly enriched in metabolic functions (p-value 1.39e-04), particularly C-compound and carbohydrate metabolism (p-value 7.74e-06) and polysaccharide metabolism (p-value 1.28e-04), but also in nitrogen, sulfur and selenium metabolism, carbohydrate transport and peptide transport. Among down-regulated genes, significant enrichment occurs for metabolism of polyketides and toxin transport [53].

The same study however also showed that in the absence of components of the heterotrimeric G-protein pathway, considerably higher differences in gene regulation between light and darkness occur, with up to 23% of differential regulation in mutants lacking the G-protein beta subunit GNB1. Thereby, a high number of genes with decreased transcript abundance in light compared to darkness was found in strains lacking PhLP1, GNB1 or GNG1. Many of these genes are not simply downregulated in light compared to the wild-type, but rather not up-regulated anymore because the light signal is not transmitted correctly. Accordingly, the highest number of consistently regulated genes in ∆*gnb1*, ∆*gng1* and ∆*phlp1* is found in the set of genes downregulated in light (628 genes including 21 glycoside hydrolase genes). This gene set includes a high number of genes associated with metabolism including 89 genes involved in C-compound and carbohydrate metabolism, but significant enrichment was only detected for functions in secondary metabolism (p-value 1.43e-08), photoperception and related functions. The strongest enrichment was observed for unclassified functions, indicating that the major function of GNB1, GNG1 and PhLP1 upon growth on cellulose still remains to be determined [53]. Available data confirm that PhLP1, GNB1 and GNG1 act in the same pathway and suggest that GNB1 and GNG1 may adjust sensitivity of the fungus to environmental signals.

Investigation of the transcriptomes of strains with mutations in the *blr1*, *blr2* or *env1* genes showed the same tendency of increased differences between transcript levels in light and darkness compared to wild-type. Light responsiveness of transcript abundances in ∆*env1* even exceeds that of ∆*gnb1* [46]. This analysis showed that the major function of BLR1 and BLR2 is observed in light, but clearly their absence did not abolish light response as the mutant strains are not blind. The majority of the regulatory targets of BLR1 and BLR2 was found in light, most of them being down-regulated in ∆*blr1* and ∆*blr2* (769 in ∆*blr1* and 873 in ∆*blr2*) and only few genes being up-regulated. In light and darkness, BLR1 and BLR2 have a considerable number of regulatory targets with major functions in C-compound and carbon metabolism, polysaccharide metabolism and transport [46]. Thereby, BLR1 and BLR2 share many targets, but in addition their function extends to individual targets as well, indicating that BLR1 and BLR2 not only act as a transcription factor complex but have individual functions as well. Independent roles have also been shown for their homologues in *T. atroviride* and *N. crassa* [47, 92]. In contrast to BLR1 and BLR2, ENV1 also exerts considerable negative effects on gene regulation in light [46].

In darkness, there are no shared targets between BLR1, BLR2 and ENV1. Only 20 genes show consistent upregulation, while 564 genes are down-regulated in light in ∆*blr1*, ∆*blr2* and ∆*env1*. These genes are dominated by functions in metabolism, including C-compound and carbohydrate metabolism including 22 glycoside hydrolase encoding genes, sulphur metabolism and with enrichment in secondary metabolism and an unexpectedly high number of genes with no assigned function.

Hence, for the photoreceptors, like for GNB1, GNG1 and PhLP1 their major function lies in up-regulation of their target genes in the presence of light, which is aimed at the light dependent modulation of metabolic functions of primary and secondary metabolism [46, 53].

Considering light dependent regulation patterns in wild-type and the available mutants in BLR1, BLR2, ENV1, GNB1, GNG1 and PhLP1, members of all glycoside hydrolase (GH) families—a remarkably high 75% of all GH encoding genes—show potential light dependent regulation.

cAMP signalling represents one of the most important output pathways of the heterotrimeric G-protein signaling cascade [95] and is hence the prime candidate for a coincidence detector between nutrient and light signaling. Transcriptome analysis of the major components of the cAMP pathway in *T. reesei*, adenylate cyclase (*acy1*) and protein kinase A (catalytic subunit 1, *pkac1*) revealed that their deletion causes an increase in light dependent gene regulation ([49]; unpublished data on ∆*pkac1*). ACY1 targets a broad array of genes involved in metabolism, Particularly, significant enrichment in metabolic functions was found for its positive regulatory targets in light [49], which is also the condition under which BLR1, BLR2, ENV1, GNB1, GNG1 and PhLP1 show their major function [49]. Comparable with ∆*acy1*, cAMP levels in ∆*env1* are very low [89]. Of the 136 positive targets of ACY1 in light, 114 overlap with those of ENV1 including 25 glycoside hydrolase encoding genes, for example *xyn2, xyn4, bxl1, cel3a, cel3b, egl1,egl2, egl6* and *cbh2,* the lytic polysaccharide monooxygenase encoding *cel61a* and *cel61b* as well as swollenin, *cip1*, *cip2* and the major transcription factor gene *xyr1*. Additionally 16 genes involved in sulphur metabolism are in this gene set [49].

These findings on altered numbers of light responsive genes lead to the conclusion that the signaling pathways for nutrient (heterotrimeric G-protein pathway, cAMP pathway) and light response balance gene expression and this balance is perturbed if single factors are removed. Since a sizable number of the targets of the investigated signaling factors show broad functions in plant cell wall degradation it can be assumed that this balance is aimed at adjustment of substrate utilization to day and night as well as growth within and on the surface of a substrate.

## Gene regulation specific for induction of cellulases is light dependent

Cellulases are expressed on diverse carbon sources, mostly representing degradation products of plant cell walls [18, 23, 96, 97]. Cellulase gene expression is not consistently regulated by light on different carbon sources [43, 65, 87, 93], indicating a different relevance of light under different conditions. Therefore, of interestis the a core gene set that is specifically regulated under inducing conditions and whether this gene set is different in light and darkness [33]. This two-dimensional analysis (5 carbon sources including inducing and repressing ones, 2 light conditions) revealed 1324 induction specific genes, comprising a set of 530 genes to be cellulase induction specific in light and in darkness, but also a considerable number of genes, that show regulation specific for cellulase induction only in light or only in darkness. The gene set of cellulase induction specific genes only in darkness includes 16 CAZyme encoding genes and comprises such important genes as *egl3* (*cel12a*), the predicted oxidoreductase TR_56840 (more than eightfold regulation in both cases), *bgl1* (*cel3a*), *egl5* (*cel45a*), *cel5b* and *bgn1* [33]. Genes of the central pathways of primary metabolism, including the pentose phosphate pathway and glycolysis turned out to be targets of light responses, with a particularly important function of ENV1. Generally, photoreceptors clearly contribute to regulation of induction specific genes both in light and darkness [33].

In several cases, specific regulation for cellulase induction [33] and positive regulation by ENV1 [46] overlaps with negative correlation of the specific protein production rate in continuous fermentations controlled for growth rate [98]. Consequently, light dependent processes and their regulation is are to be considered in fermentations as well. Of the 1324 induction specific genes, only 218 were neither light regulated nor photoreceptor targets and this gene set does not include the known cellulase regulators, glycoside hydrolases or proteins involved in oxidative degradation of cellulose [33]. Comparison of gene regulation on the insoluble cellulase inducing carbon source cellulose with the soluble inducing carbon sources lactose and sophorose [33] revealed indications that *T. reesei* may sense surfaces, particularly in light, due to cellulose specific expression of hydrophobin genes, swollenin, *cip1* and *cip2*, which play a role in the attack on cellulose [17, 99, 100]. Because of overlapping regulation with the cAMP dependent targets of ENV1 [33, 49, 89], surface sensing may be one of the outputs of the function of ENV1.

The pathways described above signal to the cell, among other information inputs, which carbon source is available in the surroundings of the cell. In this respect it is interesting that the number of light regulated genes varies on different carbon sources, like seen with different signaling mutants. In particular, the number of genes increases on lactose, sophorose and glycerol while upon growth on glucose, the number of genes down-regulated in light increases [33]. This finding explains at least in part why perturbing signaling pathways leads to altered light response: the adjustment of gene regulation according to nutrient availability does not work properly anymore.

## Genome wide transcription patterns as influenced by light

Genome wide transcriptome patterns are reflect the physiological state of the organism under the tested conditions. Therefore we wanted to assess general effects on deletion of certain signaling genes and how they relate to light response on a given carbon source. For that we re-analyzed transcriptomic data under growth on cellulose in wild-type and signaling mutants in light and darkness for the whole genome and for different gene groups [33]. Generally we found that light dependent gene regulation does not break carbon source dependent regulation [49]. This finding is also in agreement with the higher relevance of the carbon source versus the light status: in other words, light modulates gene expression, but induction or repression is not initiated or abolished by light.

Hierarchical clustering of transcript levels across different carbon sources and in the absence of nutrient and light signaling genes revealed that in mutant strains, the genome wide patterns of carbon source specific regulation are largely retained. Transcripts from cultures grown on cellulose still cluster separately from those grown on soluble carbon sources. Within soluble carbon sources i.e. comparing growth on glucose, glycerol, lactose or sophorose, also patterns on inducing and repressing carbon sources are more similar within the group than with each other. Similarly, transcript profiles from light and dark grown cultures clustered together ([33], our unpublished results). The only exception to these groupings was gene regulation in the ∆*env1* strain upon growth in light: although grown on cellulose, gene expression patterns clustered with soluble inducing carbon sources, indicating a certain malfunction in carbon sensing. Accordingly, 77% of the cAMP dependent targets of ENV1 [49] overlap with those potentially involved in cellulose/surface sensing [33].

With respect to environmental sensing, the clustering of genes encoding G-protein coupled receptors indicated perturbed carbon sensing in ∆*env1* and also in ∆*gnb1* [33]. Although deletion of *env1* causes a considerable growth defect in light [42], which is at least in part dependent on altered cAMP levels [49], deletion of *gnb1* does not result in strongly altered growth or morphology in light [53]. Consequently, this strong increase in light responsiveness of gene regulation is not simply due to altered growth in light and darkness. Moreover, altered gene regulation in light is correlating with altered growth and reflects a significant physiological modification in response to light and indicates different relevance of the targeted pathways in light and darkness.

## Non-random distribution of light regulated genes in the genome

Genes operative in the same pathway are often clustered in a genomic region, facilitating their enhanced regulation or co-regulation for example due to an open chromatin structure in this area as exemplified in secondary metabolite clusters [101]. In the genome of *T. reesei*, clustering was found for CAZyme encoding genes and in several cases genes associated with secondary metabolism were present in these genomic areas as well [102]. In *T. reesei,* light regulated genes are clustered in 15 genomic clusters (containing 66 genes) have clusters of light regulated genes when grown on cellulose, but only one cluster for growth on sophorose and 2 for growth on glucose. On lactose and glycerol no significant clusters were found [33]. In part, the light dependent clusters found on cellulose overlap with CAZyme clusters. One cluster contains the lytic polysaccharide monooxygenase encoding *cel61a* gene along with two polyketide synthase genes [33] and overlaps with a cluster positively correlated with the specific protein production rate of *T. reesei* under constant cultivation conditions [98]. This cluster represents a secondary metabolite cluster, called the “SOR cluster” which was recently characterized [54, 103]. It is responsible for production of the yellow pigment of *T. reesei* [103] and regulates biosynthesis of sorbicillin compounds [104] and their derivatives trichodimerol and dihydrotrichotetronine [54]. Interestingly, this cluster overlaps with a CAZyme cluster comprising a candidate chitinase, acetylxylanesterase *axe1*, the auxiliary protein encoding *cip1* in addition to *cel61a* [102].

The most interesting of the clusters with respect to cellulase gene regulation comprises *cbh2/cel6a*, one of the two major cellulase encoding genes of *T. reesei*, *cel5a/egl2,* a predicted sugar transporter, a putative urea transporter and an FMN dependent oxidoreductase [33]. Normal transcript levels of this cluster require the presence of BLR1, BLR2 and ENV1 in light.

Another cluster contains the mannitol dehydrogenase gene *lxr1* [105]. On mannitol, growth of the wild-type in light is slightly slower than in darkness [46]. A cluster of 9 genes around *lxr1* is regulated negatively by ENV1 in light on cellulose (up to 40 fold). This cluster overlaps with a light regulated cluster on cellulose [33, 46].

## Light influences carbon catabolite repression

Biosynthesis of enzymes is very energy consuming and hence only initiated by the fungus if the environmental conditions require these enzymes for survival [23, 34]. This is mainly the case for insoluble plant material that cannot be taken up by the cell without liberation of low molecular compounds that serve as carbon sources. The most important process for this regulation is carbon catabolite repression, which shuts down enzyme production when easily metabolizable carbon sources are sensed in the environment.

The carbon catabolite repressor CRE1 regulates cellulase gene expression not only upon growth on glucose [106], but also on cellulose [54]. Upon growth on cellulose the regulatory targets of CRE1 are different in light and darkness and few genes were consistently regulated irrespective of the light condition, and targeted functions of CRE1 are different in light and darkness as well [54]. The genes regulated by CRE1 are non-randomly distributed in the genome and form 36 clusters, several of which contain CAZyme encoding genes. Five clusters are found, of which one comprises two CAZyme encoding genes (TR_32243/carbohydrate esterase family 1 and TR_62182/glycosyltransferase family 1) that are consistently upregulated in darkness in ∆*cre1* [54]. One of the clusters shows light dependent regulation by CRE1 and photoreceptors i.e. the recently characterized SOR cluster [54, 103, 107].

Of the 1324 genes with cellulase induction specific regulation, 409 are regulated by CRE1. Of those, only 14 are downregulated in ∆*cre1* in light and darkness and only 6 (including the hydrophobin gene *hfb5*) are upregulated in light and darkness [54]. Additionally, genes regulated by CRE1 are in part non-randomly distributed in the genome. Interestingly, 142 genes are upregulated in ∆*cre1* only in light, while 251 genes are upregulated in this strain only in darkness. Consequently, the relief from carbon catabolite repression by deletion of *cre1* has also light dependent effects [54].

Among the light dependent targets of CRE1, the predicted glucose/ribitol dehydrogenase gene *grd1* was analyzed in more detail [65]. *Grd1* is positively regulated by CRE1 in darkness (more than eightfold) and its transcript size increases upon onset of inducing conditions, indicating a role of alternative splicing in its regulation. Transcript levels of *grd1* are thereby strictly correlated with those of *cbh1* and also follow the transient pattern upon sophorose induction in light [65]. GRD1 acts on cellobiose as a substrate with the likely reaction product cellobiitol, which could inhibit beta glucosidase function. With respect to cellulase regulation, GRD1 influences transcript levels, activity and protein abundance of the major cellulases in a light dependent manner. These findings suggest that GRD1 acts in intracellular sensing of cellulase efficiency and adaptation of cellulase levels for optimization of gene regulation [65].

## Regulation of plant cell wall degradation by light is conserved in fungi

After the finding that cellulases are regulated by light and photoreceptors in *T. reesei*, we were interested if this is a conserved phenomenon or specific to *Trichoderma*. Transcriptome analysis of *N. crassa* upon growth on cellulose showed that regulation of cellulases by photoreceptors is conserved [92]. Lack of photoreceptors causes initially elevated secreted cellulase activity upon growth on cellulose in *N. crassa*. After prolonged cultivation, the increased levels are only maintained in ∆*vvd*, while in the *white collar* mutants lower levels occur, which is likely due their function in regulation of energy metabolism [92]. Targets of the light response machinery in *N. crassa* include numerous genes encoding plant cell wall degrading enzymes, including several lytic polysaccharide monooxygenases as well as functions in amino acid metabolism and sulphur metabolism. 55 genes are consistently regulated in one or more of the photoreceptor mutants in *N. crassa* and *T. reesei* including the glycoside hydrolase encoding *xyn1*, *xyn3*, *cbh1* and *cbh2*. However, the important transcription factor encoding genes *xyr1*, *cre1*, *clr1* and *clr2* are not among these genes [92].

In contrast to *T. reesei,* gene regulation by the white collar proteins did not overlap with that of VVD, but indications for photoadaptation (contrasting regulation between white collars and VVD) were found. Also the striking difference of gene regulation in ∆*env1* compared to wild-type in *T. reesei* was not observed in ∆*vvd* compared to wild-type in *N. crassa* in the same extent. Targets of this photoadaptation include the carbon catabolite repressor CRE1, which is also not the case in *T. reesei*. It was particularly obvious that ribosomal genes were among those regulated by photoreceptors, which supported the hypothesis of posttranscriptional regulation of cellulase expression.

In general, this study indicates that light dependent regulation of plant cell wall degradation is a conserved process in fungi, albeit some steps of individual regulation of degradation and photoadaptation are not [92]. Exploration of light dependent gene regulation in further fungi will reveal if this conservation is indeed general.

## Light alters complex formation in promoters of cellulase genes and their regulators

Regulation of cellulase gene expression at the transcriptional level was shown to involve the CAE (*cbh2* activating element) motif in the *cbh2* promoter [108] and a similar motif in the *cbh1* promoter [109]. This DNA motif is bound by protein complexes both under inducing (sophorose) and repressing (glucose) conditions [108] and induction specific chromatin rearrangement occurs at this site [110]. Thereby, the CAE (cellulase activating element) is crucial for regulation of *cbh2* and at least in part similar proteins bind to a comparable motif in *cbh1* [111].

The findings that both *cbh1* and *cbh2* are subject to regulation by light [42, 43] indicated that complex formation within these promoters may be influenced by light. Light dependent promoter binding of the white collar complex (WCC) in *N. crassa* was shown previously and complex formation of the WCC on its target promoters is influenced by light even in cell free extracts [112].

In *T. reesei* complex formation at the CAE motif of the *cbh2* promoter is clearly different between light and darkness upon growth on lactose [87]. Additionally, one of the formed complexes at CAE is dependent on the presence of the protein kinase A catalytic subunit (PKAC1) [87].

Light dependent alterations in complex formation were also shown at the EUM1 motif, which was found as a conserved motif in the *T. reesei env1* promoter and the promoter of the *N. crassa* homologue *vvd* [42, 113]. This motif is also present in the *cbh1* and *cbh2* promoters. Cellulase gene expression is modulated by the G-protein alpha subunit GNA3 in light [77]. The EUM1 was detected in the *gna3* promoter as well. In order to investigate a possible connection or feedback between *gna3* and *env1*, we analyzed complex formation at the *gna3* promoter in darkness and after illumination upon growth on glycerol [113]. Indeed, we found light dependent changes in complex formation at the EUM1 motif in the promoters of *gna3* and *env1*. The patterns of the protein complexes strongly resembled each other in the two promoters and competition experiments confirmed that similar proteins bind to EUM1 in *gna3* and *env1* [113]. Consequently, *env1* and *gna3* are at least in part regulated by similar regulators and due to the regulation of *gna3* by ENV1 a feedback mechanism mediated by an as yet unknown transcription factor is likely.

However, the studies on complex formation at EUM1 mentioned above were done under non-inducing conditions. For growth on cellulose, complex formation also occurs within the EUM1 motifs of the *cbh2* promoter as well as on the EUM1 motifs in the *env1*, *vvd* and *gna3* promoters in a light dependent manner. Preliminary data suggest that part of the proteins binding to the EUM1 motif in these promoters also bind to the EUM1 motifs in the *env1* and in the *gna3* promoter, particularly in light (Schmoll, unpublished results).

## Carbon source utilization by *Trichoderma* is altered by light and regulated by photoreceptors

Early analysis of fungal light responses indicated that the influence of light on growth of a fungus is dependent on the carbon source it grows on [114]. Thereby, a carbon source close to its natural conditions would cause the least growth defect [115].

Using Biolog phenotype microarrays allows for simultaneous testing of growth on 95 different carbon sources [116]. Our analysis showed that there are considerable differences between growth on many of these carbon sources, particularly on substrates associated with plant cell wall degradation such as D-sorbitol, L-arabinose, d-fructose, D-galactose or xylitol [46]. Additionally, the photoreceptor ENV1 caused significantly different growth patterns on many carbon sources [45]. Correlating these results with gene regulation data from transcriptome analyses showed that the pentose-phosphate pathway responsible for utilization of substrates like xylose or lactose, is a target of light response and subject to regulation by photoreceptors. At several of the enzymatic steps in this pathway, modification of growth correlated with light dependent alteration of enzyme gene transcript levels [46].

In addition, the positive regulator of cellulase gene expression, GRD1, influences light dependent growth patterns, especially on several intermediates of lactose and D-galactose carabolism, like D-xylose, D-galactose, d-fructose, D-mannitol and D-xylose. This influence on growth on intermediates of a cellulase inducing carbon source (lactose) is in agreement with the function of GRD1 in cellulase regulation and potentially inducer formation [65].

In *T. atroviride*, stimulation of growth in light was observed on many carbon sources, particularly 17 carbon sources which are well utilized by this fungus [117]. These carbon sources comprise common building blocks of plant cell walls like fructose, mannose, galactose, glucose or xylose, while growth on the best carbon source for *T. atroviride*, glycerol, is not influenced by light and growth on sorbitol even decreased in light. The increase in growth rate on several carbon sources in light is dependent on the presence of BLR1 and BLR2. Lack of these factors even causes decreased growth, albeit some exceptions on certain carbon sources for ∆*blr1* and ∆*blr2* confirm that these strains are not totally blind, but rather show altered and diminished responses [117].

The clear connection between the cAMP pathway and cellulase regulation by light and photoreceptors indicated a role of BLR1 and BLR2 in introducing the light signal into the cAMP pathway upon regulation of utilization of diverse carbon sources. In *T. atroviride* stimulation of growth by addition of cAMP was observed only on a few carbon sources (glucose, gentiobiose, cellobiose and xylose) and this response was dependent on BLR1 and BLR2. On these carbon sources, stimulation of growth was also achieved by addition of menadione that causes oxidative stress, which should simulate oxidative stress caused by light. Also for this response functional BLR1 and BLR2 are required. However, a general correlation of responses to cAMP and oxidative stress with the presence or absence of BLR1 or BLR2 across all 95 carbon sources tested could not be confirmed [117].

Consequently, light influences growth and carbon utilization of *T. atroviride* and the cAMP pathway as well as oxidative stress response are involved in this regulation mechanism. Additionally, BLR1 and BLR2 play important roles in this connection particularly on carbon sources representing building blocks of plant cell walls, hence confirming carbohydrate degradation as a major target of light response.

## Crucial factors for plant cell wall degradation as targets of light response

The long history of research towards improvement of cellulase gene expression yielded a considerable number of regulators enhancing or decreasing efficiency of plant cell wall degradation [23, 31]. Transcriptome analysis showed that carbon metabolism and degradation of polysaccharides are important targets of light dependent gene regulation in *T. reesei* and *N. crassa* [53, 92]. For *N. crassa* it has been shown that the photoreceptors themselves act on regulation of further transcription factors, constituting a flat hierarchy of regulation [118]. Consequently, the transcription factors involved in plant cell wall degradation are of particular interest as targets of light response.

Therefore we screened the recently published, comprehensive list of transcription factors involved in plant biomass utilization [31] for genes differentially regulated in light and darkness or by photoreceptors. Interestingly, we found that among them, the transcript levels of *xyr1*, *ace1*, *ace2*, *ace3*, *vib1* and *amyR* are decreased in light compared to darkness in QM6a upon growth on cellulose (Table 1). Also the *T. reesei* homologues of the important *N. crassa* transcription factors *clr*-*1* and *clr*-*2* show regulation in QM6a by light, but they do not regulate cellulase expression in *T. reesei* ([119] and our unpublished results). *Xyr1* is regulated by BLR1 and ENV1. Interestingly, the mating type protein MAT1-2-1 interacts with XYR1 and is recruited to the *cbh1* promoter in a XYR1 and light dependent manner [120]. The finding that ENV1 considerably regulates *mat1*-*2*-*1* [44], the hypothesis that this light dependent effect is due to the function of photoreceptors is supported. *Ace3*, *vib1* and *amyR* are regulated by ENV1 upon growth on cellulose (Table 1; [46]).

**Table 1:** Light dependent regulation of transcription factors involved in plant biomass utilization

Name	Protein ID	Regulatory function (related to plant biomass degradation) in	Cellulase induction specific			Regulation in QM6a in light	Regulated by BLR1	Regulated by BLR2	Regulated by ENV1
			In light	In darkness	In light and darkness				
XYR1	122208	(hemi-)cellulose utilization	x	x	x	--	x		x
CRE1	120117	Carbon catabolite repression				n			
ACE1	75418	Cellulose utilization				-			
ACE2	78445	Cellulose utilization		x		--			
ACE3	77513	Cellulose utilization	x	x	x	---			x
CLR1	27600	(hemi-)cellulose utilization (in *N. crassa*)		x		-			x
CLR2	26163	(hemi-)cellulose utilization (in *N. crassa*)				--	x		x
XPP1	122879	(hemi-)cellulose utilization				n			
VIB1	54675	Cellulose utilization				---			x
HAP2	124286	CAZy regulation				n			
HAP3	121080	CAZy regulation				n			
HAP5	124301	CAZy regulation				n			
AmyR	55105	Starch utilization				--			x
MalR	21997	Maltose utilization				n			
BglR	52368	Sugar sensing				n			
RhaR1	79871	L-rhamnose utilization				n			
RhaR2	121107	L-rhamnose utilization	x	x	x	n			
McmA	42249	Cellulase regulation				n			
VEL1	122284	Cellulase regulation				++			
VEL2	40551	Metabolism				n			
AreA	76817	N-assi mi lation				n			
AreB	120127	Nitrogen metabolite repression				n			
NmrA	74375	Nitrogen metabolite repression				n			
NIT4	76705	Nitrate pathway				n			
PAC1	120698	pH response				n			
PACX	59740	pH response				-			

For the regulatory impact of the GATA-type transcription factors BLR1 and BLR2 see text on photoreceptors

n, no regulation; +, upregulation in light; -, downregulation in light; +/-, minor regulation; ++/--, moderate regulation (around 5–10 fold); +++/---, strong regulation

A further important parameter for plant cell wall degradation is the hydrolysis performance as determined by the secretome of *T. reesei* [121]. This study identified 12 proteins to be limiting for hydrolysis. Ten of them were found to be regulated in a cellulase induction specific manner in light or darkness or both [33] (Table 2). But most importantly, all of the genes encoding these limiting proteins were strongly downregulated in light in QM6a upon growth on cellulose and regulated by ENV1 under the same conditions. Additionally, all those genes except *xyn4* are regulated by BLR1 and *man1*, *xyn2* and *bgl1* are regulated by BLR2 as well [46].

**Table 2:** Light dependent regulation of genes encoding proteins limiting for hydrolysis of pretreated corn stover

Name	Protein ID	Function	Cellulase induction specific			Regulation in QM6a in light	Regulated by BLR1	Regulated by BLR2	Regulated by ENV1
			In light	In darkness	In light and darkness				
	69276	Xylanase	x	x	x	---	x		x
CIP1	73638	Carbohydrate binding module containing	x	x	x	---	x		x
CIP2	123940	Glucuronyl esterase				---	x		x
MAN1	56996	Beta-mannanase	x	x	x	---	x	x	x
EGL2	120312	Endoglucanase CEL5A	x	x	x	---	x		x
XYN2	123818	Xylanase	x	x	x	---	x	x	x
EGL3	123232	Endoglucanase CEL12A		x		---	x		x
SWO1	123992	Swollenin	x	x	x	---	x		x
BGL1	76672	Beta-glucosidase		x		---	x	x	x
CBH2	72567	Cellobiohydrolase CEL6A	x	x	x	---	x		x
EGL6	49081	Xyloglucanase CEL74A				---	x		x
XYN4	111849	Xylanase	x	x	x	---			x

n, no regulation; +, upregulation in light; -, downregulation in light; +/-, minor regulation; ++/--, moderate regulation (around 5–10 fold); +++/---, strong regulation

These findings are in perfect agreement with polysaccharide degradation being a major target of light response in *T. reesei* [53]. Moreover, these results indicate that for stable and predictable performance of strains in research and industry, light conditions and random light pulses have to be considered of equal importance as for example pH and oxygen supply and a defined cultivation medium.

## Implications for research, industrial application and strain improvement

Fungi sense their environment and adjust growth and enzyme production for efficient use of resources accordingly. They use complex signal transduction pathways to integrate these environmental signals for a defined response to the conditions they perceive—in their natural environment as well as in an industrial setting. Consequently, knowledge on signal transduction pathways is highly relevant for efficient and stable gene expression [122].

In order to draw reliable conclusions on gene regulation, we have to ensure that the growth conditions of the organisms we are testing remain to same. Reproducibility of results requires defined growth media in terms of carbon source, nitrogen source, pH, temperature etc. Often, different standard media and growth conditions for different fungi are discussed as problematic for comparability of data. The research reviewed here clearly shows that light is an important environmental cue not only in *T. reesei* where light can alter regulation of cellulase genes tenfold and more. Nevertheless, even in *T. reesei* there is still research towards gene regulation and cellulase gene expression performed under random light conditions. With transcriptional regulation, it is known that even a few seconds of light cause a response in gene regulation. Considering the broad metabolic regulations by light, this compares to using a random mixture of carbon sources in an assay, which may show a beneficial effect first and no effect at all upon repetition. Moreover, we recently showed that cellulase regulation additionally involves a posttranscriptonal section, which is triggered by the light sensitive heterotrimeric G-protein pathway [33]. Consequently, good laboratory practice has to include the use of controlled light conditions in order to draw any reasonable conclusions as to the function of a gene. Especially for transcriptome studies, the effect of random light pulses—for example during harvesting or due to an open shaker—can be dramatic and lead to wrong conclusions on the function of a gene in enzyme production.

The implementation of such data from uncontrolled laboratory experiments into industrial strain improvement may hence lead to costly failures either early in testing or later in upscaling. For example, if constitutive activation of the G-protein alpha subunit GNA3 [77] would be tested for improvement of cellulase fermentations: Shake flask cultivations on table top shakers in the lab (in light) and subsequently in a glass fermentor would lead to beneficial effects on cellulase expression, because GNA3 exerts its function in light. However, upon (cost intensive) upscaling in a dark steel fermentor, the strain would not show benefits anymore, because in darkness constitutive activation of GNA3 does not have an effect on cellulase regulation [77]. Research done under random conditions bears such costly risks for surprises, because based on current knowledge the difference in the function of a given regulator in light and darkness cannot be predicted reliably. Only if part of the pathway is already known, like for the heterotrimeric G-protein pathway, some perspectives can be suggested.

### Exploitation of light dependent effects

Available data show that enzyme production is most efficient in darkness in *T. reesei*. Nevertheless, the strong differences between light and darkness indicate an efficient regulatory mechanism, that—due to previous negligence of light dependent phenomena—bears the chance to identify novel regulators that can be artificially misregulated for dark specific cellulase enhancement in an industrial fermentor (Fig. 2).

Since light does not override the induction process [33, 49], the use of photofermentors for enzyme production in a mass fermentation is unlikely to be cost effective. However, for special high value products like secondary metabolites, the use of light dependent promoters for precisely triggered regulation should be kept in mind. Similarly, secondary metabolism can be altered by application of light which might be beneficial for co-production or production of valuable metabolites. Additionally, the striking differences between light and darkness can be used to select light responsive promoters to enable triggering or abolishing protein production using light pulses. Exemplary studies in yeast [123] and *T. reesei* [124] have already proven that this approach is successful.

Besides the actual production process, also inoculum production is crucial for stable and high level production. Production of spores is regulated by light in fungi. Moreover, conditions of spore production are crucial for physiology of the next generation of fungi grown from it, particularly with respect to stress response. Accordingly, our own research showed that reproducible results require constant conditions for inoculum generation (precultivation on plates) prior to cultivation in liquid culture and analysis (Schmoll and Tisch, unpublished). Although a circadian rhythmicity has not yet been demonstrated for *T. reesei*, our experience strongly suggests its operation. Therefore, random light pulses, which reset the molecular circadian clock should be avoided during spore production to ensure reliable production in the cultivation thereafter. It remains to be determined whether constant light or constant darkness or light cycles of defined length during preculture can be used to increase production capacity for fermentations.

## Conclusions

Since the discovery that light influences regulation of cellulases and more generally carbohydrate active enzymes, several regulation mechanisms were shown to govern this influence. In some cases, regulators only show an effect under one light status but not another. Growth, enzyme regulation, carbon utilization, carbon catabolite repression and even cross talk with secondary metabolism and sulfur metabolism were shown. Therefore, in order to obtain scientifically meaningful results on gene regulation aimed at investigation of plant cell wall degradation, application of controlled light conditions is mandatory and even short random light pulses are to be avoided.
